# Sustainability of UK shale gas in comparison with other electricity options: Current situation and future scenarios

**DOI:** 10.1016/j.scitotenv.2017.11.140

**Published:** 2018-04-01

**Authors:** Jasmin Cooper, Laurence Stamford, Adisa Azapagic

**Affiliations:** School of Chemical Engineering and Analytical Science, The University of Manchester, The Mill, Room C16, Sackville Street, Manchester M13 9PL, UK

**Keywords:** Shale gas, Fracking, Hydraulic fracturing, Electricity, Sustainability, Multi-criteria decision analysis

## Abstract

Many countries are considering exploitation of shale gas but its overall sustainability is currently unclear. Previous studies focused mainly on environmental aspects of shale gas, largely in the US, with scant information on socio-economic aspects. To address this knowledge gap, this paper integrates for the first time environmental, economic and social aspects of shale gas to evaluate its overall sustainability. The focus is on the UK which is on the cusp of developing a shale gas industry. Shale gas is compared to other electricity options for the current situation and future scenarios up to the year 2030 to investigate whether it can contribute towards a more sustainable electricity mix in the UK. The results obtained through multi-criteria decision analysis suggest that, when equal importance is assumed for each of the three sustainability aspects shale gas ranks seventh out of nine electricity options, with wind and solar PV being the best and coal the worst options. However, it outranks biomass and hydropower. Changing the importance of the sustainability aspects widely, the ranking of shale gas ranges between fourth and eighth. For shale gas to become the most sustainable option of those assessed, large improvements would be needed, including a 329-fold reduction in environmental impacts and 16 times higher employment, along with simultaneous large changes (up to 10,000 times) in the importance assigned to each criterion. Similar changes would be needed if it were to be comparable to conventional or liquefied natural gas, biomass, nuclear or hydropower. The results also suggest that a future electricity mix (2030) would be more sustainable with a lower rather than a higher share of shale gas. These results serve to inform UK policy makers, industry and non-governmental organisations. They will also be of interest to other countries considering exploitation of shale gas.

## Highlights

• Shale gas ranks between the fourth and eighth relative to other electricity options.
• To become the most sustainable option, large improvements would be needed.
• This includes a 329-fold reduction in environmental impacts.
• A 16-fold increase in employment would also be needed.
• An electricity mix with less rather than more shale gas is more sustainable.

## Graphical abstract

**Figure:**
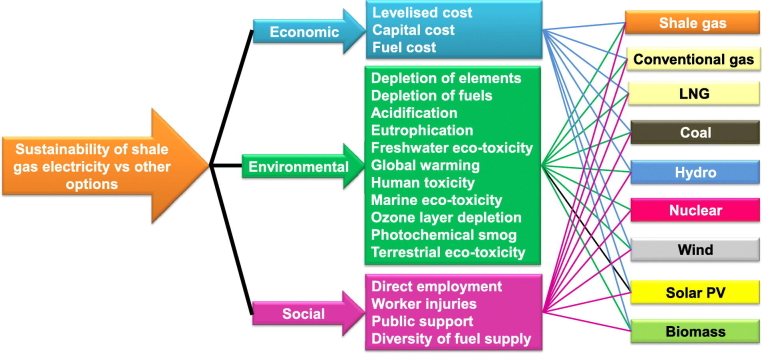

## 1 Introduction

Exploitation of shale gas is a contentious topic in many countries. At present, shale gas is exploited at a large scale only in the US, with other nations considering its development (Cooper et al., 2016). The UK is at the cusp of starting exploitation, with the government and industry keen to develop a shale gas industry, but with a strong opposition from numerous stakeholders, including non-governmental organisations, local residents and activists (Gosden, 2017, Johnston, 2017, Ward, 2017). The impacts on the environment are the main argument against the exploitation of shale gas while the supporters highlight improved national energy security and economic development as key aspects in its favour (House of Lords, 2014, Moore et al., 2014). Some of these sustainability aspects have been considered previously by the authors (Cooper et al., 2014, Cooper, 2017), but evaluated environmental, economic and social aspects in isolation of each other. This work builds on that research by integrating all three dimensions to assess the overall sustainability of shale gas in the UK using multi-criteria decision analysis (MCDA). The main goals of this study are:

i) to assess the overall sustainability of shale gas relative to other electricity options in the UK, including other fossil alternatives, renewables and nuclear power; and
ii) to investigate how its deployment could affect the sustainability of a future UK electricity mix, taking into account different levels of shale gas penetration.

In total, 18 sustainability indicators are considered, of which 11 are environmental, three economic and four social. While there have been numerous other studies on the sustainability of shale gas, they are almost exclusively based in the US and tend to focus on environmental aspects, typically considering only one or a limited number of impact categories; for an extensive review, see Cooper et al. (2016). Therefore, as far as we are aware, this is the first study internationally to provide an integrated assessment of shale gas and to compare it other electricity options.

The methods used in the study are outlined in the next section. The results are presented and discussed in Section 3 and conclusions are drawn in Section 4.

## 2 Methods

The environmental and economic sustainability assessments have been carried out using life cycle assessment (LCA) and life cycle costing (LCC), respectively; social sustainability has been evaluated by developing relevant social sustainability indicators. A brief overview of these is given below, followed by a description of the MCDA method used.

### 2.1 Sustainability assessment

The results of the LCA, LCC and social sustainability assessment are summarised in Table 1, based on the previous work by the authors (Cooper et al., 2014, Cooper, 2017); for definitions of the indicators, see Table S1 in the Supporting Information (SI). In addition to shale gas, the following electricity options are also considered: conventional gas, liquefied natural gas (LNG), coal, nuclear, hydro, wind, solar photovoltaics (PV) and biomass. These options have been chosen as they are currently used in the UK and are expected to play a role in a future electricity mix.

**Table 1:** Sustainability indicators and their estimated values for different electricity options^a^.

Sustain-ability aspects	Indicators	Shale gas	Conven'l gas	Liquefied natural gas	Coal	Nuclear	Hydro	Solar PV	Wind	Biomass
Environmental^b^	ADP_e_ (mg Sb_-Eq._/kWh)	0.68	0.24	0.26	0.04	0.07	0.01	10.91	0.22	0.14
	ADP_f_ (MJ/kWh)	6.58	6.33	7.43	11.70	0.09	0.04	1.05	0.15	0.62
	AP (g SO_2-Eq._/kWh)	0.35	1.71	3.41	5.13	0.06	0.01	0.43	0.06	1.39
	EP (g PO_4-Eq._/kWh)	0.17	0.06	0.06	1.86	0.02	0.01	0.29	0.03	0.49
	FAETP (g DCB_-Eq_./kWh)	13.10	2.47	4.02	287.90	21.20	1.65	63.90	14.70	20.90
	GWP (g CO_2-Eq._/kWh)	455.78	420.00	490.00	1078.84	7.79	3.70	88.91	12.35	58.51
	HTP (g DCB_-Eq._/kWh)	54.30	38.00	39.50	294.86	111.43	6.15	205.47	61.81	208.50
	MAETP (kg DCB_-Eq._/kWh)	37.42	0.50	0.90	1577.32	43.66	2.70	205.69	23.08	42.48
	ODP (μg R11_-Eq._/kWh)	17.30	18.90	5.51	5.59	19.00	0.23	17.40	0.74	5.16
	POCP (mg C_2_H_4-Eq._/kWh)	83.80	34.40	66.60	285	5.55	2.04	67.00	6.97	131
	TETP (g DCB_-Eq._/kWh)	1.70	0.15	0.22	1.75	0.74	0.19	1.12	1.81	4.26
Economic	Levelised cost of electricity (pence/kWh)	9.59	8.00	7.62	13.85	7.70	14.60	6.70	9.73	11.75
	Capital cost (pence/kWh)	0.81	0.90	0.81	4.60	7.00	11.29	5.70	7.70	4.50
	Fuel cost (pence/kWh)	6.51	4.90	4.53	3.60	0.50	0.00	0.00	0.00	5.30
Social	Direct employment (person-yr/TWh)	47.70	62.00	326.88	191.00	87.00	782.35	653.00	368.00	385.79
	Worker injuries (no. injuries/TWh)	0.53	0.54	2.10	4.50	0.59	14.59	4.84	2.30	2.98
	Public support index (%)	5.60	34.00	14.50	− 7.00	9.00	72.00	75.00	59.00	57.00
	Diversity of fuel supply (no units)	1.00	1.00	0.04	0.86	0.85	1.00	1.00	1.00	0.96

a Data for the environmental indicators sourced from Cooper et al. (2014) and the economic and social from Cooper (2017).

b For the acronyms, see the caption for Fig. 1.

Both the current electricity mix and future scenarios are considered. As commercial production of shale gas is not expected in the UK until post-2020 (Lewis et al., 2014), the year 2030 has been selected for the evaluation of a future electricity mix. Two 2030 electricity scenarios are considered: one with low penetration of shale gas (1%) and another with the highest possible contribution (8%) to the mix; for details, see Table 2. The results of the LCA, LCC and social sustainability assessment for the current and future electricity mixes are given in Table 3 (Cooper et al., 2014, Cooper, 2017).

**Table 2:** Current electricity mix and future scenarios^a^.

Electricity source	Current situation (2012) (TWh)	2030 (low shale penetration) (TWh)	2030 (high shale penetration) (TWh)
Shale gas	0.00	4.60	28.74
Conventional gas	83.53	67.82	67.82
Liquefied natural gas	14.67	28.74	4.60
Coal	136.00	18.51	18.51
Nuclear	63.90	101.85	101.85
Hydro	5.28	8.51	8.51
Solar PV	1.19	3.00	3.00
Wind	19.58	104.68	104.68
Biomass	15.20	35.00	35.00

a Coal and gas carbon capture and storage are included in the 2030 mix, assuming an equal split between coal and gas.

**Table 3:** Sustainability indicators and their estimated values for the current electricity mix and future scenarios (Cooper et al., 2014, Cooper, 2017).

Aspects	Indicators	Current mix (2012)	2030 (low shale gas penetration)	2030 (high shale gas penetration)
Environmental^a^	ADP_e_ (mg Sb_-Eq_./kWh)	0.08	0.24	0.27
	ADP_f_ (MJ/kWh)	6.44	3.05	3.01
	AP (g SO_2_-_Eq._/kWh)	2.24	0.48	0.48
	EP (g PO_4_-_Eq._/kWh)	0.77	0.11	0.12
	FAETP (g DCB_-Eq._/kWh)	118.84	17.56	18.04
	GWP (kg CO_2-Eq._/kWh)	560	150	150
	HTP (g DCB_-Eq_./kWh)	143.64	92.84	93.64
	MAETP (kg DCB_-Eq_./kWh)	62.34	7.94	8.09
	ODP (μg R11_-Eq_./kWh)	12.41	13.67	14.30
	POCP (mg C_2_H_4-Eq._/kWh)	137.93	45.64	46.56
	TETP (g DCB_-Eq._/kWh)	1.25	1.44	1.52
Economic	LCOE (pence/kWh)	9.72	10.99	10.95
	Capital cost (pence/kWh)	3.22	5.27	5.27
	Fuel cost (pence/kWh)	2.86	2.13	2.26
Social	Direct employment (person-yr/TWh)^b^	175.30	233.04	214.96
	Worker injuries (injuries/TWh)^b^	2.65	1.95	1.85
	Public support index (%)	15.23	33.66	33.08
	Diversity of fuel supply (−)	0.89	0.90	0.93

a For the acronyms, see the caption for Fig. 1.

b Data not available for coal and gas carbon capture and storage.

### 2.2 Multi-criteria decision analysis

The Simple Multi-attribute Rating Technique (SMART) method has been chosen for the MCDA in this work because it is relatively simple to implement and can accommodate a large number of criteria and alternatives being considered. SMART involves the following steps (Edwards, 1977):

1. identification of the options to be compared;
2. identification of the decision criteria;
3. scoring of the criteria in the order of importance (increasing from a score of 10 for the lowest importance onwards) and estimation of their weights of importance;
4. rating of the options on a scale of 0 (worst) to 1 (best);
5. estimation of the overall scores and ranking of the options on a scale from 0 (worst) to 1 (best); and
6. identification of the best option.

The MCDA has been carried out using the Web-HIPRE tool (Mustajoki and Hamalainen, 2000) based on the decision tree in Fig. 1. Following the SMART methodology, the sustainability aspects and indicators have been weighted based on their assumed relative importance and the options rated based on their performance for each indicator (see Table 1) using value factions. Two types of value functions - linear and exponential - have been applied to investigate the effect on the overall ranking of the options and gauge the robustness of the results. The calculated weightings and ratings have then been used to estimate the overall sustainability score - the option with the highest value is considered the most sustainable and vice versa. For further details on the SMART methodology, see Section S1 in the SI.

**Fig. 1:**
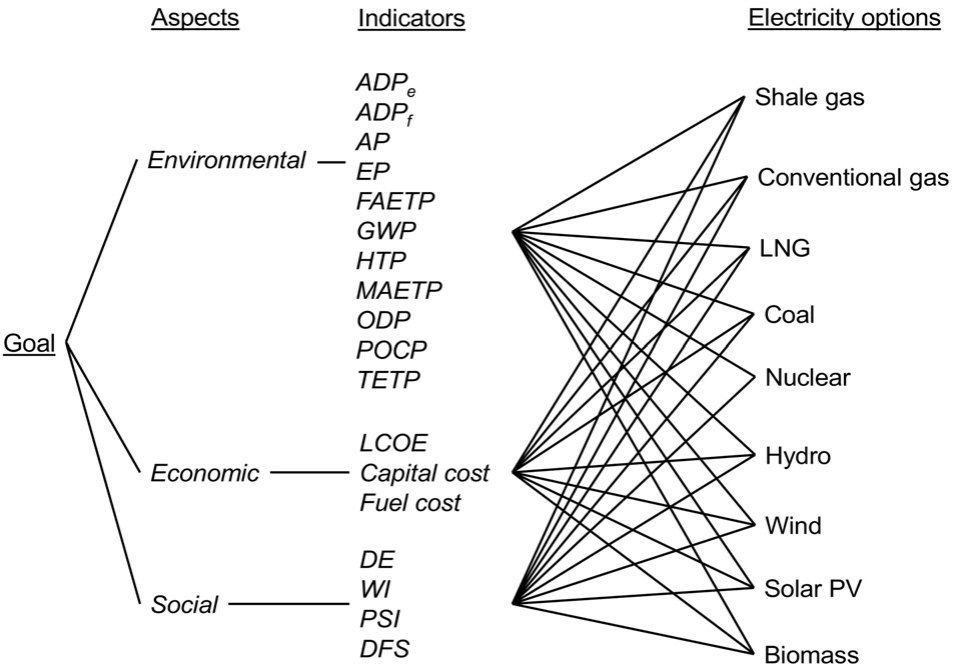
MCDA decision hierarchy, showing the sustainability aspects, indicators and electricity options considered in the analysis. [Goal: i) to assess the overall sustainability of shale gas relative to the other electricity options in the UK; ii) to find out how its deployment could affect the sustainability of a future UK electricity mix. Indicators: ADP_e_: abiotic depletion of elements; ADP_f_: abiotic depletion of fossil fuels; AP: acidification potential; EP: eutrophication potential; FAETP: freshwater aquatic ecotoxicity; GWP: global warming potential; HTP: human toxicity potential; MAETP: marine aquatic ecotoxicity potential; ODP: ozone depletion potential; POCP: photochemical oxidant creation potential; TETP: terrestrial ecotoxicity potential; LCOE: levelised costs of electricity; DE: direct employment; WI: worker injuries; PSI: public support index; DFS: diversity of fuel supply].

Two MCDA models have been constructed in Web-HIPRE, one comparing shale gas with the other electricity options and another comparing the present and future electricity mixes. The former is based on the data summarised in Table 1; the data for the second MCDA model can be found in Table 2, Table 3.

In the base case, it is assumed that all three sustainability aspects (environmental, economic and social) are equally important, assigning each a weighting of 0.33; the effects of changing the importance of the aspects have been assessed through extensive sensitivity analyses. A further analysis has also been carried out to find out to what extent the weightings would need to change for shale gas to emerge as the most sustainable option overall, or to be comparable with conventional gas, LNG, renewables or nuclear power. The required improvements in the performance of shale gas for different sustainability indicators have also been considered.

### 2.3 Data quality assessment

A data quality assessment has been carried out to evaluate the overall quality of the data used in the study and, through that, the validity of the results. A pedigree matrix, typically used in LCA (Althaus et al., 2007, Weidema et al., 2013), has been applied for these purposes. The pedigree matrix rates data quality on the following six criteria on a scale from 1 (high) to 5 (low): reliability, completeness, temporal correlation, geographical correlation, technological correlation and sample size. For further details, see Table S2 in the SI.

The data have been rated for each of the above criteria and averaged for each sustainability aspect, using the results from LCA, LCC and social sustainability assessment, respectively. The ratings have then been added up to calculate the overall data quality score for each sustainability aspect, ranging between 6 and 30 as follows:

• 6 to 12: high quality;
• > 12 to 18: medium quality;
• > 18 to 24: medium-low quality; and
• > 24: low quality.

## 3 Results

This section first compares the overall sustainability of shale gas with the other electricity options. This includes a sensitivity analysis and the improvements in the life cycle of shale gas electricity that would be required to improve its overall ranking. This is followed by a comparison of the current electricity mix with the future scenarios and, finally, by the assessment of data quality.

### 3.1 Sustainability of shale gas compared to other electricity options

**Fig. 2:**
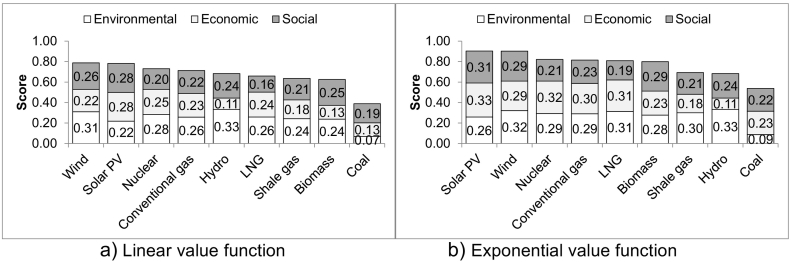
Ranking of the electricity options assuming equal weightings for the sustainability aspects and indicators.

The results in Fig. 2 indicate that, if the environmental, economic and social aspects are equally important, the best options are wind and solar PV with scores of 0.79 and 0.78 (linear value function; LVF) and 0.90 (exponential; EVF) while the worst is coal with 0.39 (LVF) and 0.54 (EVF). Shale gas ranks seventh out of nine options for both value functions, scoring 0.64 and 0.69, respectively. The best and the worst options are unaffected by the type of the value function used but the order of some other options changes. For example, hydroelectricity ranks fifth for the LVF and eighth for the EVF, while biomass ranks eighth for the LVF and sixth for the EVF. This is because the LVF does not take into account the magnitude of the difference in values of different indicators (for these, see Figs. S1 and S2 in the SI). For instance, while biomass scores poorly for six out of 11 environmental indicators and for two out of three economic indicators, it is still much better (up to two orders of magnitude) than the worst option for each indicator (see Table 1). Thus, using the EVF, which takes this into account, is arguably more appropriate.

As can be seen in Fig. 2, the environmental aspect contributes the most towards the overall score for shale gas (38% for the LVF and 43% for the EVF), followed by the social (30% and 33%) and finally the economic aspect (26% and 29%). Similar contributions are found for most other options. The exception is coal where the social dimension is dominant (41% and 48%) and the environmental has a small influence overall (16% and 18%).

### 3.2 Sensitivity analysis

The sensitivity analysis explores how the ranking of the options changes when one of the three sustainability aspects is prioritised over the other two. In each case, the weightings for each aspect have been changed in turn until the ranking of the best or worst option changed. The equal importance of each sustainability indicator remains unchanged throughout. These results are discussed in turn in the next sections.

#### 3.2.1 Environmental aspect

**Fig. 3:**
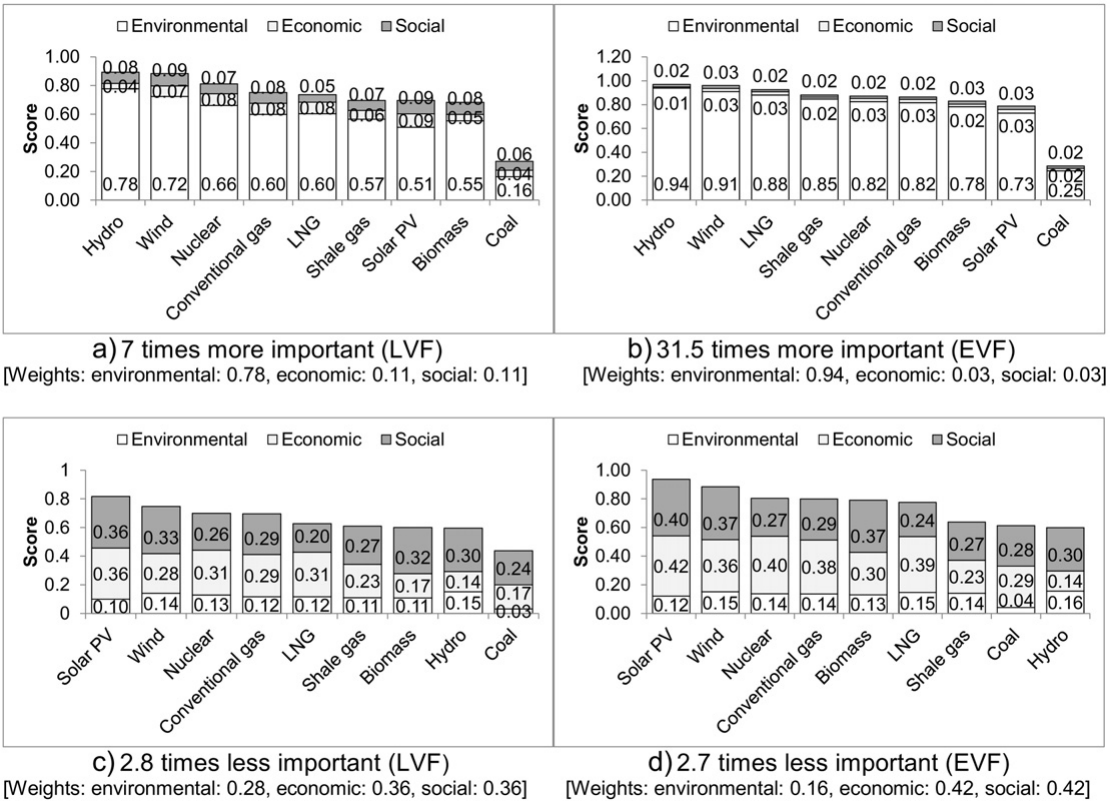
Ranking of the electricity options assuming different importance of the environmental aspect.

If the environmental aspect is assumed more important than the other two, wind and solar PV remain the best options until the weighting for the environmental aspect is seven times higher for the LVF and 31.5 times for the EVF (Fig. 3a&b). At and above these weightings, hydropower is the most sustainable option, followed closely by wind while solar PV drops to the seventh (LVF) and eighth place (EVF). Shale gas is ranked, respectively, sixth, followed closely by solar PV, and fourth, being only marginally better than nuclear and conventional gas. For both value functions and all the options, the main contributor to the overall sustainability score is the environmental aspect, which is to be expected given its high assumed importance.

When the importance of the environmental aspect is reduced by 2.8 times for the LVF, solar PV outranks wind as the best option and shale gas is ranked sixth, marginally better than biomass and hydroelectricity (Fig. 3c). For the EFV (Fig. 3d), the importance of this aspect has to be 2.7 times lower than of the other two dimensions of sustainability for the rankings to change; solar PV is still the best option but hydro is now the least sustainable, together with coal and followed closely by shale gas in the seventh place. In both cases, the economic and social aspects have a similar contribution, dominating the overall sustainability scores, while the contribution of the environmental aspect is small, again as expected, given its assumed low priority.

#### 3.2.2 Economic aspect

**Fig. 4:**
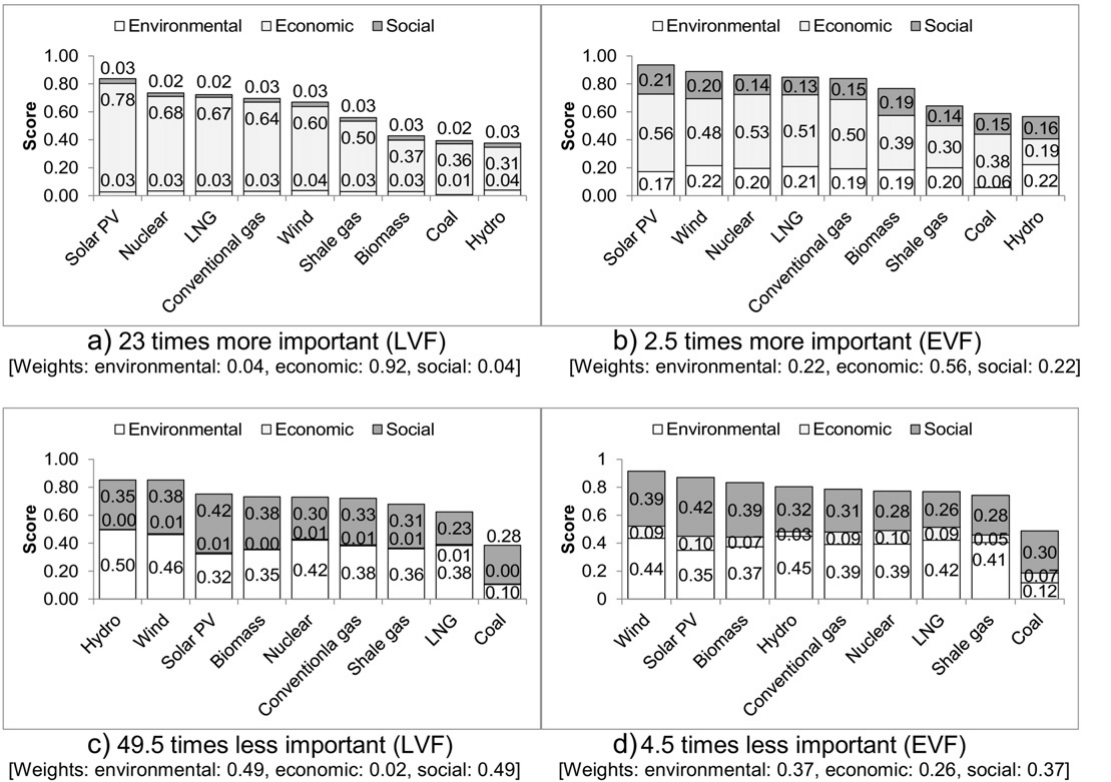
Ranking of the electricity options assuming different importance of the economic aspect.

Wind and solar PV remain the best options until the weighting of the economic aspect is 23 times higher for the LVF and 2.5 times for the EVF (Fig. 4a&b). At and above these weightings, solar PV is still the best option but hydro becomes the least sustainable, followed closely by coal. It is interesting to note that for the LVF, wind drops to the fifth place (Fig. 4a) because of its poor performance in levelised and capital costs. Shale gas is ranked sixth for the LVF and seventh for the EVF.

On the other hand, if the economic aspect is assumed to be the least important, the rankings remain the same until it is 49.5 times less important. In that case, hydro is the most sustainable option jointly with wind for the LVF (Fig. 4c); shale gas is in the seventh place. For the EVF, wind overtakes solar PV as the most sustainable option when the importance of the economic aspect is reduced by 4.5 times (Fig. 4d).

#### 3.2.3 Social aspect

**Fig. 5:**
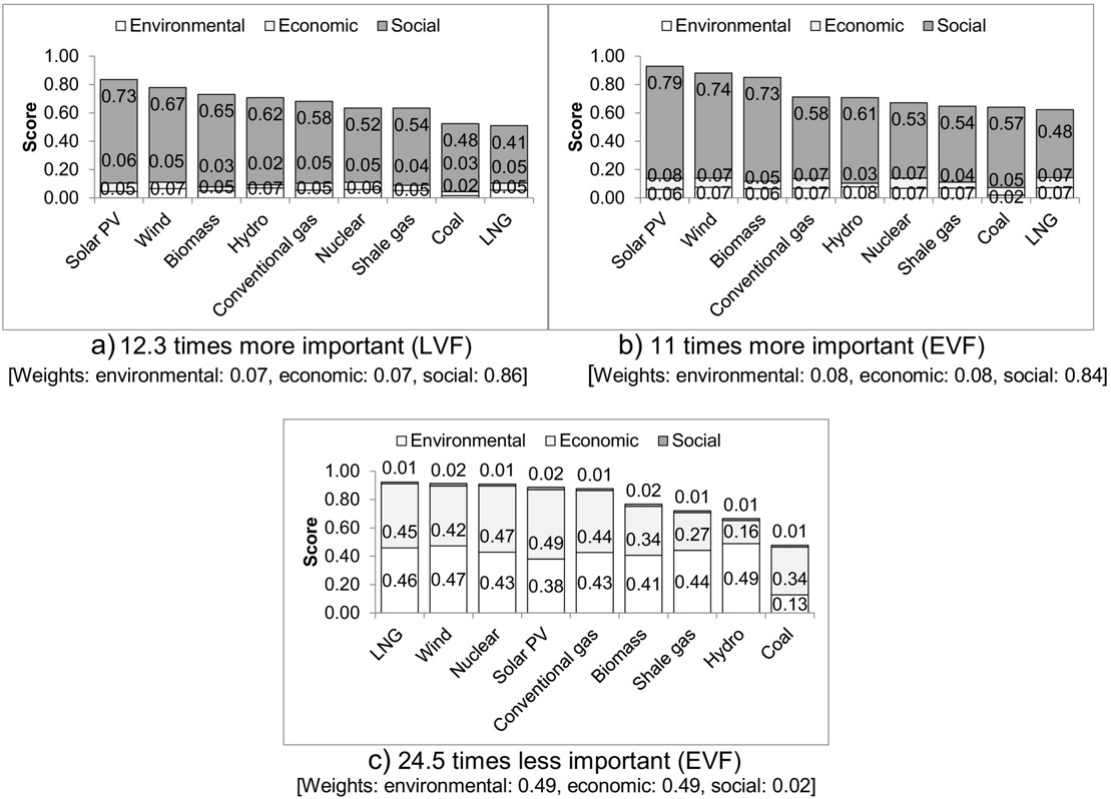
Ranking of the electricity options assuming different importance of the social aspect.

The ranking of the options changes when the social aspect is 12.3 times more important than the other two for the LVF and 11 times for the EVF at which point LNG becomes the least sustainable option, narrowly behind coal (Fig. 5a&b). Shale gas ranks seventh for both value functions, following nuclear power; for the EVF, it is only marginally better than coal and LNG.

When the importance of the social aspect is reduced by 24.5 times for the EVF, LNG becomes the most sustainable option, being only slightly better than wind, nuclear, solar PV and conventional gas (Fig. 5c). Coal remains the least sustainable option and shale gas is ranked seventh. For the LVF, there is no change in the rankings with a reduction in the importance of the social aspect.

### 3.3 Changes needed for shale gas to become the most sustainable option

This section aims to determine what would be required for shale gas to become the most sustainable option among those considered in this study. First, multiple indicators are considered simultaneously for each sustainability aspect before looking at the individual indicators.

#### 3.3.1 Multiple sustainability indicators and aspects

Based on its performance in different sustainability aspects and indicators (Table 1), and not considering any improvements in the sustainability, there are only two scenarios in which shale gas would become the top-ranking option (jointly with some others). These are as follows:

1. joint best with LNG and conventional gas if the capital cost is 1000–10,000 times more important than the other economic indicators and, simultaneously, the economic aspect is 1000 times more important than the other two aspects; and
2. joint best with conventional gas and nuclear if the importance of worker injuries is 1000 times higher than of the other social indicators and, at the same time, the importance of the social aspect is 1000 times greater than of the other two.

For the remaining indicators, shale gas can never be the best option unless its performance is improved considerably. For example, a 40%–70% improvement is needed in all the indicators for shale gas to become the most sustainable option, jointly with wind and solar PV (Table 4). If the performance is improved in one sustainability aspect at a time, even larger improvements are needed. For the environmental aspect, a 100-fold reduction in the environmental impacts is required and this aspect has to be 3.8–23 times more important than the other two. For the economic aspect, a large reduction (50% to 20 times) in costs is needed, together with an increase in the importance of this aspect (up to 2.5 times) for shale gas to be the best option, together with solar PV. However, for greater reductions to all cost indicators (see Table 4), no changes in the importance of the economic aspect are needed. Improvements in the social indicators are only applicable to employment and public support, which must be improved by at least 16 and 13 times, respectively, for shale gas to emerge as the top option (Table 4). Thus, based on these results, it is highly unlikely that shale gas would be the most sustainable option among those assessed here.

**Table 4:** Improvements needed in different sustainability aspects and indicators for shale gas to be the most sustainable option.

Scenario	Improvements needed for shale gas to become the best option^a^	Notes^a^
Improvements in all indicators	Improvements of 45% (LVF) and 70% (EVF) in all indicators; equal weighting for all three aspects and indicators	EVF: For a 40% improvement in all indicators and the economic aspect eight times more important, shale gas is the best option with solar PV
Improvements in the environmental indicators only	100-fold reduction in all environmental impacts for both value functions; equal weighting for environmental indicators, but environmental aspect must be 3.8 times more important than the economic and social aspects for the LVF and 23 times more important for the EVF	Marginally better than wind assuming equal importance of the aspects. At a ten-fold reduction for both value functions, equal indicator weightings and 10,000 times greater importance of the environmental aspect, shale gas is marginally worse than the best option (hydro)
Improvements in the economic indicators only	Five times reduction in all cost indicators and no change in the importance of the aspects for it to be the best option together with solar PV (LVF). A 20-fold reduction in costs with the economic aspect 1.5 times more important to be marginally better than wind and solar PV (EVF)	LVF: At 50% reduction in all costs and economic aspect 2.5 times more important, shale gas is the best option, marginally better than solar PV. For the reduction in LCOE of 30% and zero fuel cost, shale gas is the best option together with wind assuming equal importance of all three sustainability aspects. EVF: For the 4.8 times lower LCOE and zero fuel cost, shale gas is the best option with wind and solar PV assuming equal importance of the sustainability aspects.
Improvements in the social indicators only	> 13 times better PSI^a^ and > 16 times greater DE^a^ for both value functions	Improvements are only applicable to PSI and DE as shale gas is the best option for WI and scores the maximum for DFS.

a LVF: linear value function; EVF: exponential value function; LCOE: levelised cost of electricity; DE: direct employment; PSI: public support index; DFS: diversity of fuel supply.

#### 3.3.2 Individual sustainability indicators and aspects

The results of the analysis when considering improvements in one indicator at a time together with its related aspect are shown in Table 5. As can be seen, the environmental impacts would need to be reduced by 9–329 times and their importance would have to be 10,000 times higher than of the other indicators, together with a 100 times greater importance of the environmental aspect relative to the other two. For the economic indicators, the levelised cost of electricity (LCOE) needs to be reduced by 32%, with its importance increasing by 100 times, together with a similar increase in the importance of the economic aspect. As mentioned in the previous section, the capital cost does not need to be reduced, but if it is, then its importance must be increased by up to 10,000 times relative to the other indicators, along with a 100–10,000 times higher importance of the economic aspect (Table 5). The social indicators would need improvements similar in magnitude to those needed for the environmental indicators: direct employment by 16.4 times and public support by 13.6 times. Worker injuries do not need to be reduced for the ranking to change, but this indicator must be considered 1000–10,000 times more important than the others, together with a similar increase in the importance of the social aspect over the other two.

**Table 5:** Improvements needed in each indicator for shale gas to be the most sustainable option (changing one indicator at a time)^a^.

Indicators	Current values	Improved values	Units	Increase in the indicator importance	Increase in importance of related aspect^b^	Notes
ADP_e_	0.68	0.007	mg Sb_-Eq._/kWh	10,000	100	Shale gas the best option, jointly with hydro
ADP_f_	6.58	0.02	MJ/kWh	10,000	100	Shale gas the best option, jointly with hydro
AP	0.35	0.011 g	g SO_2-Eq._/kWh	10,000	100	Shale gas the best option, jointly with hydro
EP	0.17	0.0045	g PO_4-Eq._/kWh	10,000	100	Shale gas the best option, jointly with hydro
FAETP	13.10	1.4	g DCB_-Eq._/kWh	10,000	100	Shale gas the best option, jointly with hydro
GWP	455.78	3	g CO_2-Eq._/kWh	10,000	100	Shale gas the best option, jointly with hydro
HTP	54.30	5.9	g DCB_-Eq._/kWh	10,000	100	Shale gas the best option, jointly with hydro
MAETP	37.42	0.49	kg DCB_-Eq._/kWh	10,000	1000	. Shale gas the best option, jointly with conventional gas and LNG
ODP	17.30	0.21	μg R11_-Eq._/kWh	10,000	100	Shale gas the best option, jointly with hydro
POCP	83.80	1.8	mg C_2_H_4-Eq._/kWh	10,000	100	Shale gas the best option, jointly with hydro
TETP	1.70	0.14	g DCB_-Eq._/kWh	10,000	100	Shale gas the best option, jointly with conventional gas
LCOE	9.59	6.50	Pence/kWh	100 (LVF) 100 (EVF)	10 (LVF) 100 (EVF)	Shale gas the best option, jointly with solar PV
Capital cost	0.81	0.80 (LVF) 0.65 (EVF)	Pence/kWh	1000 (LVF) 10,000 (EVF)	100 (LVF) 10,000 (EVF)	Shale gas the best option, jointly with LNG
Fuel cost	6.51	0	Pence/kWh	10,000	10,000	Shale gas the best option, jointly with hydro, wind and solar PV
Direct employment	47.70	783	Person-yr/TWh	100 (LVF) 1000 (EVF)	100 (LVF) 10 (EVF)	Shale gas the best option, jointly with conventional gas
Worker injuries	0.53	0.53	Injuries/TWh	1000 (LVF) 10,000 (EVF)	1000 (LVF) 1000 (EVF)	Shale gas the best option, jointly with conventional gas
Public support index	5.60	76	%	1000	1000 (LVF) 100 (EVF)	Shale gas the best option, jointly with solar PV
Diversity of fuel supply	1.00	1.00	–	10,000	10,000	Shale gas the best option, jointly with conventional gas, hydro, wind and solar PV

a For the acronyms, see the caption in Fig. 1. The values for the environmental indicators are the same for the linear value function (LVF) and exponential value function (EVF) as are the weightings required. Differences between LVF and EVF for the economic and social indicators are noted in the table where relevant.

Therefore, the above results suggest that shale gas is unlikely to be the best option in comparison to the other alternatives considered in this work as large improvements and considerable, sometimes extreme, increase in the importance of indicators and aspects would be needed.

### 3.4 Changes needed for shale gas to be comparable to different electricity options

The results in the previous section demonstrate that it is all but impossible for shale gas to be considered the most sustainable option. While this is informative, arguably, it is not necessary for shale gas to be the most sustainable option and it could still potentially be viable if it can compete with some of the other established electricity options. Therefore, this section considers what would be needed to achieve that, starting with the focus on other fossil fuels (conventional gas and LNG), followed by nuclear and finally the renewable options (hydroelectricity and biomass). Wind and solar PV are not considered as they are the most sustainable options based on the results discussed in 3.1, 3.2 so that improvements needed for shale gas to compete with these two would be similar to those considered in the previous section. Coal is not considered either as shale gas is a more sustainable option for most scenarios discussed in the previous sections. For brevity, only an overview of the results is provided here; for the detailed discussion, see Section S4 in SI.

#### 3.4.1 Comparison of shale gas with conventional gas and LNG

As conventional gas and LNG rank higher than shale gas in the base case (Section 3.1), large reductions in the impacts of shale gas are needed, along with changes in the importance of the sustainability aspects. As indicated in Table 6, the environmental and social aspects need significant improvements while only a moderate reduction is needed for the economic costs. For example, for the LVF, to be comparable with conventional gas, a 20% reduction in environmental impacts from shale gas is necessary and the environmental aspect must be 13 times more important than the other two. Alternatively, 80% reduction in impacts should be achieved if all three aspects are considered equally important. For the EVF, a 100-fold reduction in environmental impacts is needed and the aspect must be three times more important. Similar results are found for LNG assuming the EVF.

**Table 6:** Improvements needed for each sustainability aspect for shale gas to become comparable to conventional gas and LNG.

Sustainability aspect	Conventional gas		LNG	
	Linear value function	Exponential value function	Linear value function	Exponential value function
Environmental	20% reduction in environmental impacts and aspect 13 times more important or 80% reduction in impacts and equal importance as the other two aspects	100-fold reduction in environmental impacts and aspect three times more important	20% reduction in environmental impacts and aspect 13 times more important or 40% reduction in impacts and equal importance as the other two aspects	100-fold reduction in environmental impacts and aspect 5.2 times more important
Economic	30% reduction in costs and equal importance as the other two aspects	25% reduction in costs and equal importance as the other two aspects	10% reduction in costs and equal importance as the other two aspects	20% reduction in costs and equal importance as the other two aspects
Social	Five-fold increase in DE^a^ and PSI^a^ and aspect 4.7 times more important or ten-fold increase in DE and PSI and equal importance as the other two aspects	Five-fold increase in DE^a^ and PSI^a^ and aspect 4.7 times more important	Five-fold increase in D^a^E and PSI^a^ and equal importance as the other two aspects	Ten-fold increase in DE^a^ and PSI^a^ and equal importance as the other two aspects

a DE: direct employment; PSI: public support index.

When the individual indicators are considered, reductions are needed in nine out of 11 environmental impacts for shale gas to compete with conventional gas and in eight relative to LNG. Improvements are also needed in social and economic indicators: 18%–31% for LCOE and fuel cost and 32% to 6.5-fold for direct employment and public support (Table S2), along with large increases in the importance of these indicators (100–1000) and their related sustainability aspects (10–100 times). For the remaining indicators, no improvements are needed, but unlike the environmental indicators, large increases in aspect/indicator importance are needed for fuel cost and diversity of fuel supply (100–10,000 times).

#### 3.4.2 Comparison of shale gas with nuclear power

As nuclear power ranks significantly better than shale gas in the base case (Fig. 2), significant improvements and increases in the importance of sustainability aspects and indicators are needed if shale gas is to be comparable. The magnitude of the improvements and increases in importance are similar to those needed for it to compete with conventional gas as nuclear has a similar ranking to it (Fig. 2). As shown in Table 7, the environmental and social aspects need the largest improvements (up to 100 times) while the needed reductions in costs are smaller (25%–40%).

**Table 7:** Improvements needed for each sustainability aspect for shale gas to become comparable to nuclear power.

Sustainability aspect	Nuclear power	
	Linear value function	Exponential value function
Environmental	Five-fold reduction in environmental impacts and aspect 1.8 times more important or 100-fold reduction in environmental impacts and equal importance as the other two aspects	100-fold reduction in environmental impacts and aspect 3.3 times more important
Economic	30% reduction in costs and aspect three times more important or 40% reduction in costs and equal importance as the other two aspects	25% reduction in costs and equal importance as the other two aspects
Social	Ten-fold increase in DE^a^ and PSI^a^ and aspect 1.3 times more important or 13-fold increase in DE and PSI and equal importance as the other two aspects	Ten-fold increase in DE^a^ and PSI^a^ and aspect 1.5 times more important or 17-fold increase in DE and PSI and equal importance as the other two aspects

a DE: direct employment; PSI: public support index.

When each indicator is targeted individually (Table S3), improvements are needed in seven out of the 11 environmental indicators. For these, 89% to 91-fold reductions are needed along with large increases in aspect/indicator importance (100–10,000 times). For the economic and social indicators, improvements are needed in the levelised cost of electricity, fuel cost, direct employment and public support (Table S3). The levelised costs of electricity need a 21% reduction and 10–100 times increase in aspect/indicator importance, while the fuel cost must be reduced 16-fold and the importance of the aspect and the indicator should increase by 100–1000 times. A 72%–79% increase in direct employment and public support are required along with a 100-fold increase in the aspect and indicator importance (Table S3).

#### 3.4.3 Comparison of shale gas with hydro and biomass electricity

As both hydro and biomass electricity are closer in ranking to shale gas than conventional gas, LNG and nuclear, smaller improvements and increases in the importance of the aspects and indicators are needed, as shown in Table 8. The social aspect needs the largest improvement (8–10 times), followed by the environmental (20%–50%) and economic (20%) aspects.

**Table 8:** Improvements needed for each sustainability aspect for shale gas to become comparable to hydro and biomass electricity.

Sustainability aspect	Hydro	Biomass
	Linear value function	Exponential value function
Environmental	50% reduction in environmental impacts and equal importance as the other two aspects	20% reduction in environmental impacts and aspect 3.9 times more important
Economic	20% reduction in costs and equal importance as the other two aspects	20% reduction in costs and equal importance as the other two aspects
Social	Eight-fold increase in DE^a^ and PSI^a^ and equal importance as the other two aspects	Ten-fold increase in DE^a^ and PSI^a^ and equal importance as the other two aspects

a DE: direct employment; PSI: public support index.

However, significant improvements (9–329 times) are needed in all environmental indicators for shale gas to compete with hydroelectricity (Table S4). A 100–10,000 times increase in the importance of the aspect and the indicators is also needed. For the economic indicators, shale gas has lower levelised and capital cost than both hydro and biomass electricity, but its fuel cost is higher. As a result, no reductions in levelised and capital cost are needed but an increase in aspect/indicator importance of up to 1000 times is required (Table S4). On the other hand, fuel cost must be reduced to zero and the importance of the aspect/indicator increase 10,000-fold for it to compete with hydroelectricity while a 20% reduction and a 100-fold increase in the importance is needed for it to compete with biomass.

No improvement in worker injuries is needed but up to 50-fold increase in aspect/indicator importance is required. Direct employment should be improved by 16.4 times and 13 times higher public support is required for shale gas to compete with hydropower, along with a 100-fold increase in aspect/indicator importance (Table S4). To compete with biomass, an eight-fold increase in direct employment and 10.4 times greater public support are needed, together with 100–1000 times increase in aspect/indicator importance. For the diversity of fuel supply, biomass scores lower than shale gas and hence no improvements are needed, but a five to 100 times increase in aspect/indicator importance is necessary.

### 3.5 Effect of shale gas on the sustainability of electricity generation

The results in Table 9 suggest that, assuming equal importance of all the sustainability aspects and indicators, the electricity mix with low penetration of shale gas (1% on the grid) is considerably more sustainable than for the higher contribution (8%), with the respective sustainability scores of 0.74 and 0.44. This is to be expected because, as discussed in the previous sections, shale gas generally scores poorly in various impacts, including global warming potential, fuel cost and public support (see Table 1). Only when a 10,000 lower importance is placed on the environmental aspect do the two electricity mixes become comparable (Table 9).

**Table 9:** Sustainability scores for the low and high penetration of shale gas into the 2030 electricity mix in comparison with the current mix, assuming differing importance of sustainability aspects.

Importance of aspects	Current situation (2012)	2030 (low penetration of shale gas)^a^	2030 (high penetration of shale gas)^a^
Equal importance	-b	0.74	0.44
Environmental aspect 10,000 times less important than economic and social	-b	0.67	0.67
Equal importance	0.39	0.69	0.63
Economic aspect 3.9 times more important than the other two	0.53	0.52	0.46

a Low penetration: 1% contribution to the electricity mix. High penetration: 8% contribution.

b No values as the comparison is between future electricity mixes only.

When the individual indicators are considered, for the high-penetration electricity mix to become comparable with the low, improvements are necessary in all but two environmental impacts as well as in capital and fuel costs, employment and public support. As can be seen in Table 10, the improvements needed range from 2%–16%. However, no changes to the importance of any aspect are required, except for the social when considering diversity of fuel supply; however, the importance assigned to each indicator must increase five to 80-fold.

**Table 10:** Improvements needed for each indicator for the 2030 high shale contribution mix and present mix to become comparable to the low shale contribution mix^a^.

Indicator^b^	Units	High shale gas contribution				Current situation (2012)			
		Current values	Improved values	Increase in importance of indicator	Increase in importance of related aspect	Current values	Improved values	Increase in importance of indicator	Increase in importance of related aspect
ADP_e_	mg Sb_-Eq._/kWh	0.27	0.24	11	–	0.08	0.08	20	–
ADP_f_	MJ/kWh	3.01	3.01	15	–	6.44	2.90	20	–
AP	g SO_2-Eq._/kWh	0.48	0.46	11	–	2.24	0.46	15	–
EP	g PO_4-Eq._/kWh	0.12	0.10	11	–	0.77	0.10	30	–
FAETP	g DCB_-Eq._/kWh	18.04	17.50	11	–	118.84	17.50	1000	3
GWP	g CO_2-Eq._/kWh	150	150	15	–	560	150	1000	–
HTP	g DCB_-Eq._/kWh	93.64	92.00	11	–	143.64	92.00	100	–
MAETP	kg DCB-_Eq._/kWh	8.09	7.93	11	–	62.34	7.93	1000	5
ODP	μg R11_-Eq._/kWh	14.30	13.60	11	–	12.41	12.41	30	–
POCP	mg C_2_H_4-Eq._/kWh	46.56	45.00	11	–	137.93	45.00	100	–
TETP	g DCB_-Eq._/kWh	1.52	1.43	11	–	1.25	1.25	30	–
LCOE	pence/kWh	10.95	10.95	22	–	9.72	9.72	2	3.5
Capital cost	pence/kWh	5.27	5.26	5	–	3.22	3.22	2	3.5
Fuel cost	pence/kWh	2.26	2.12	10	–	2.86	2.12	2	3
Direct employment	person-yr/TWh	214.96	234.00	5	–	175.30	234	20	10
Worker injuries	injuries/TWh	1.85	1.85	10	–	2.65	1.84	15	3
Public support index	%	33.08	34.00	5	–	15.23	34.00	20	20
Diversity of fuel supply	–	0.93	0.93	80	8	0.89	0.94	80	30

a Results shown are for the linear value function only for illustration as the choice of the value function does not affect the ranking.

b For the acronyms, see the caption in Fig. 1.

It can also be seen in Table 9 that both 2030 electricity mixes are more sustainable than the present mix, assuming equal importance of all three sustainability aspects. This is not because of shale gas but due to a large drop in the contribution from coal and growth in renewables. The current mix is only better if the economic aspect is 3.9 times more important than the other two. This is due to the average levelised cost of fossil fuels being lower than that of renewables, making 2030 electricity more expensive. For the current electricity to be comparable to the 2030 mixes, improvements must be made to all social indicators (6% to 2.2 times), eight environmental impacts (36% to 7.9-times) and fuel cost (26%); see Table 10. An increase in the importance of the indicators and their related aspects (up to 1000-fold) is also required.

### 3.6 Robustness analysis

To assess the robustness of the results with respect to the MCDA method and the weightings used, the same analysis has been performed using direct weighting (DW) (Mustajoki and Hamalainen, 2000) as an alternative. This method is similar to SMART except that the weightings are inputted directly into the model, while in SMART they are calculated based on the assigned scores (see Section 2.1 and Section S1 in the SI). The rankings obtained through DW remained the same for all the weightings considered in SMART and discussed in the previous sections, thus validating the robustness of the results. It is possible that the rankings would change with other MCDA methods but their exploration is out of the scope of this paper.

### 3.7 Data quality

As discussed in Section 2.3, the quality of the data underlying the sustainability assessment has been evaluated according to the six criteria in the pedigree matrix (see Table S2 in the SI). Overall, the data quality is estimated to be ‘medium’ for the environmental and economic assessments, ‘high’ for the social sustainability assessment and ‘medium’ for the 2030 electricity mix (Table 11). This would suggest that the results are valid, but further improvements to the data used would increase their robustness.

**Table 11:** Data quality assessment using a pedigree matrix.

	Environmental data (LCA)^a^	Economic data (LCC)^a^	Social data (SSA)^a^	2030 Electricity mix
Reliability	3.06	2.17	1.36	1.88
Completeness	1.25	1.00	1.04	1.00
Temporal correlation	1.26	1.08	1.44	1.75
Geographical correlation	1.80	1.00	2.37	1.47
Technological correlation	1.75	1.83	1.52	1.03
Sample size	4.70	5.00	3.71	5.00
Overall data quality	13.82 (Medium)	12.08 (Medium)	11.44 (High)	12.13 (Medium)

a LCA: life cycle assessment; LCC: life cycle costing; SSA: social sustainability assessment.

Some data sources were of poor quality, in particular the sample size for the LCC data and geographical correlation for the LCA (Table S3 in SI). This is due to the data used to estimate the cost of producing shale gas in the UK being based on reports which estimate the cost of establishing a UK shale industry. Similarly, as the UK has no shale gas industry but only exploration wells, US data for material and process requirements have been used to model shale gas wells. Despite this, the overall data quality is ‘medium’ to ‘high’. Also, the quality of the literature data scored well in comparison to the Ecoinvent data used (Table S3 in SI).

## 4 Conclusions

The results of this study show that, assuming equal importance of the environmental, economic and social aspects, shale gas ranks seventh out of the nine electricity options considered for both values functions. In that case, wind and solar PV are the most sustainable and coal is the worst option. If the environmental impacts are the most important, hydropower becomes the best option, with shale gas ranking fourth to seventh, depending on the value function used. For high importance of the economic aspect, solar PV is the best option while coal and hydropower represent the least sustainable options; shale gas ranks sixth or seventh. Finally, if social aspect is the most important, solar PV is the most sustainable option with coal and LNG being the worst options; shale gas is in the sixth to eighth place. Therefore, while overall not the worst, shale gas is not one of the better options either.

Despite this, it is possible to arrive at an outcome where shale gas is the best option by altering the importance placed on the indicators and aspects, as well as by improving its performance in different indicators. However, these are very significant and unrealistic. For example, if the importance of the capital cost and the economic aspect is 10,000 higher, shale gas becomes the best option (together with conventional gas and LNG). Similarly, when the importance of worker injuries and the social aspect is increased 10,000-fold, shale gas emerges as the most sustainable option, along with conventional gas. However, for the other indicators, large improvements would be needed in combination with very significant increases in the importance placed on the sustainability aspects and indicators.

For the environmental aspect, improvements in impacts can lead to shale gas becoming the best option (jointly with hydro) but only at a 9–329 fold reduction and in combination with significant increases in their importance (100–10,000 times). For the economic aspect and indicators, the levelised cost must be reduced by a minimum of 32% and their importance must be increased by 10–100 times. Alternatively the fuel cost must be zero and its importance increased by 10,000-fold for shale gas to be the most sustainable option, together with hydro, wind and solar PV. Large increases in the importance (100–1000 times) are also required for public support and employment, together with improvements in their values (13.6 and 16.4 times, respectively). No improvements are necessary for diversity of fuel supply but the importance of this indicator and the social aspect must increase 10,000-fold and even then it is level with conventional gas, hydro, wind and solar PV as the most sustainable options.

To be comparable with conventional gas, LNG and nuclear power, large improvements in the performance of shale gas are needed, along with significant increases in the importance of the sustainability aspects and indicators. For example, to compete with nuclear power, an 89% to 91-fold reduction in environmental impacts is needed and their importance must be increased by 100–1000 times. However, this is only applicable to seven out of the 11 indicators. For the remaining four, no improvements are needed as shale gas has lower impacts, but a 5–1000 times increase in their importance is necessary. In some scenarios, shale gas is already more sustainable than hydro and biomass, but in others, large improvements to environmental and social impacts would be needed.

The results also suggest that a future electricity mix with a lower penetration of shale gas is more sustainable than the one with higher contribution, assuming the sustainability aspects are of equal importance. If higher importance is placed on the economic or social aspect, the high shale gas mix outranks the low due to the relatively low cost of shale gas compared to renewables.

Although the quality of the data used in this study is considered ‘medium’ to ‘high’, some data are derived from non-UK sources, which is one of the limitations of this work. If or when the exploitation of shale gas starts in the UK, using actual field data would help to refine the findings of this research. A further limitation is the limited number of economic and social indicators considered and future work should consider others, such as tax revenue, contribution to gross domestic product, community benefits, local employment, noise and traffic, to name a few. Another limitation is a lack of stakeholder input into the decision analysis, particularly their preferences for different sustainability aspects and indicators. Despite the study showing that the overall conclusions are robust to changes in preferences, future work should consider involving relevant stakeholders and exploring the effect of their actual preferences on the outcomes of the sustainability assessment. Future work could also explore the effect on the sustainability of shale gas of different technological solutions. These include techniques for disposal of drilling waste and wastewater treatment, 'green completion' and carbon capture, storage and/or utilisation.

While this study focused on the UK, the methodological approach is generic enough to be applicable to sustainability evaluations of shale gas in other countries and regions - this is recommended as part of future research to help decision-makers make more informed decisions. It would also help to find out if the results for different regions (e.g. Europe) can be generalised to guide future policy development related to shale gas.

## Appendix A Supplementary data

Supplementary information contains: description of the SMART method; definition of sustainability indicators used in the study; variations in sustainability indicators for different electricity options; changes needed in each indicator for shale gas to be comparable to other electricity options; data pedigree matrix; and data quality results. Image 1

## References

[bb0005] Althaus H.-J., Doka G., Dones R., Heck T., Hellweg S., Hischier Roland, Nemecek T., Rebitzer G., Spielmann M., Wernet G. (2007). Overview and Methodology: Data v2.0 (2007). http://www.ecoinvent.org/database/older-versions/ecoinvent-version-2/methodology-of-ecoinvent-2/methodology-of-ecoinvent-2.html.

[bb0010] Cooper J. (2017). Life Cycle Sustainability Assessment of Shale Gas in the UK. PhD Thesis.

[bb0015] Cooper J., Stamford L., Azapagic A. (2014). Environmental impacts of shale gas in the UK: current situation and future scenarios. Energ. Technol..

[bb0020] Cooper J., Stamford L., Azapagic A. (2016). Shale gas: a review of the economic, environmental, and social sustainability. Energ. Technol..

[bb0025] Edwards W. (1977). How to use multiattribute utility measurement for social decisionmaking. IEEE Trans. Syst. Man Cybern..

[bb0030] Gosden E. (2017). Cuadrilla starts drilling at Lancashire fracking site. The Times, 18 August 2017.

[bb0035] House of Lords (2014). The Economic Impact on UK Energy Policy of Shale Gas and Oil. May, 2014. http://www.publications.parliament.uk/pa/ld201314/ldselect/ldeconaf/172/172.pdf.

[bb0040] Johnston I. (2017). Election 2017: conservatives back fracking 'revolution' in the party manifesto. The Independent 18 May 2017.

[bb0045] Lewis C., Speirs J., MacSweeney R. (2014). Getting ready for UK shale gas: Supply chain and skills requirements and opportunities. United Kingdon Onshore Oil and Gas and Ernst and Young.

[bb0050] Moore V., Bereford A., Gove B., Underhill R., Parnham S., Crow H., Cunningham R., Huyton H., Sutton J., Melling T., Billings P., Salter M. (2014). Hydraulic Fracturing for Shale Gas in the UK: Examining the Evidence for Potential Environmental Impacts. https://www.rspb.org.uk/Images/shale_gas_report_evidence_tcm9-365779.pdf.

[bb0055] Mustajoki J., Hamalainen R.P. (2000). Web-HIPRE: global decision support by value tree and AHP analysis. Information Systems and Operational Research (INFOR).

[bb0060] Ward A. (2017). Ineos wins injunction against shale protesters. Financial Times (FT), 31 July 2017.

[bb0065] Weidema B.P., Bauer C., Hischier R., Mutel C., Nemecek T., Reinhard J., Vadenbo C.O., Wernet G. (2013). Overview and Methodology: Data Quality Guideline for Ecoinvent Database Version 3 (Final). https://www.ecoinvent.org/files/dataqualityguideline_ecoinvent_3_20130506.pdf.

