# Supplementary material for: Sustainability of UK shale gas in comparison with other electricity options: Current situation and future scenarios
Source: Sci Total Environ. 2018 Apr 1;619-620:804–14. doi: 10.1016/j.scitotenv.2017.11.140 (PMC5895563; doi:10.1016/j.scitotenv.2017.11.140)
Supplement: Supplementary file 1 — Supplementary information contains: description of the SMART method; definition of sustainability indicators used in the study; variations in sustainability indicators for different electricity options; changes needed in each indicator for shale gas to be comparable to other electricity options; data pedigree matrix; and data quality results. [file mmc1.pdf]

Supporting information

Sustainability of UK shale gas and other electricity options: current situation and future scenarios

Jasmin Cooper, Laurence Stamford, Adisa Azapagic

List of contents

S1. The SMART method
S2. Sustainability indicators
S3. Variations in sustainability indicators across the electricity options
S4. Changes needed in each indicator for shale gas to be comparable to other electricity options
S5. Pedigree matrix
S6. Data quality results
References

S1. The SMART method

The relative importance of a sustainability aspect or indicator is represented by its weighting. The weighting is calculated relative to the other aspects/indicators in the same category. To calculate the weighting, each aspect or indicator is first scored, with a score of 10 indicating the least important aspect/indicator and the more important ones assigned higher scores. The scores for all the aspects or indicators in a given category are then summed up and the weighting of each aspect and indicator calculated according to eqns. (S1) and (S2), respectively:

$$W_a = \frac{I_a}{\sum_a^A I_a} \quad \text{(S1)}$$

$$W_b = \frac{I_b}{\sum_b^B I_b} \quad \text{(S2)}$$

where:

W_a the weighting of sustainability aspect *a* (environmental, economic or social)
I_c the importance of aspect a
a sustainability aspect
A total number of aspects
W_b the weighting of sustainability indicator b
I_b the importance of indicator b
b sustainability indicator
B total number of indicators.

The alternatives considered, in this case different electricity options, are rated based on their performance in each indicator and the ratings are calculated using a value function. The worst performing option is given a rating of zero and the best a rating of one. The remaining options are rated between these two values, in the order of their performance. The ratings vary depending on the type of value function used. A linear value function assumes that the changes from worst to best are linear. However, it does not take into consideration large gaps in between. Non-linear value functions can also be used when the distribution of scores is not even, or there are outliers or gaps in the distribution. An exponential value function using the bisection method was selected for this work. In this method, the difference between the worst and the bisection point is of equal importance to the difference between the bisection point and the highest value, enabling consideration of large gaps.

The overall sustainability score is calculated for each option based on the estimated weightings of the sustainability aspects and indicators and the ratings of the options, as follows:

$$S_o = \sum_a^A W_a \times \left(\sum_b^B W_b \times R_{o,b}\right) \quad \text{(S3)}$$

where:

S overall sustainability score
R_b rating of option *o* for indicator b

The alternatives are then ranked, with the one with the highest score being the most sustainable and the one scoring the lowest, the least sustainable.

S2. Sustainability indicators

Table S1: Description of indicators used in the MCDA

Indicator	Units[a]	Description
Abiotic depletion of elements *(ADP_e)*	kg $Sb_{-Eq.}$/kWh	Potential for the depletion of elemental metals and minerals e.g. gold, phosphorus and copper
Abiotic depletion of fossil fuels *(ADP_f)*	MJ/kWh	Potential for the depletion of fossil fuels
Acidification potential *(AP)*	kg $SO_{2-Eq.}$/kWh	Potential for acid deposition and formation in water and terrestrial ecosystems
Eutrophication potential *(EP)*	kg $PO_{4-Eq.}$/kWh	Potential for over-fertilisation of aquatic and terrestrial ecosystems
Freshwater aquatic ecotoxicity *(FAETP)*	kg $DCB_{-Eq.}$/kWh	Potential for the release of substances toxic to freshwater water environments
Global warming potential *(GWP)*	kg $CO_{2-Eq.}$/kWh	Potential for the release of greenhouse gases
Human toxicity potential *(HTP)*	kg $DBC_{-Eq.}$/kWh	Potential for the release of substances toxic to human health into the environment
Marine aquatic ecotoxicity potential *(MAETP)*	kg $DCB_{-Eq.}$/kWh	Potential for the release of substances toxic to marine water environments
Ozone depletion potential *(ODP)*	kg $R11_{-Eq.}$/kWh	Potential for emissions of ozone depleting substances
Photochemical oxidant creation potential *(POCP)*	kg $C_2H_{2-Eq.}$/kWh	Potential for the creation of photochemicals
Terrestrial ecotoxicity potential *(TETP)*	kg $DCB_{-Eq.}$/kWh	Potential for the release of substances toxic to terrestrial environments
Levelised cost of electricity *(LCOE)*	p/kWh	The ratio of total financial inputs required to generate electricity to the total amount of electricity generated by the power plant
Capital cost	p/kWh	The ratio of the total capital required to build a power plant to the total amount of electricity generated
Fuel cost	p/kWh	The ratio of the total cost for fuel incurred by a power plant to the total amount of electricity generated
Direct employment *(DE)*	person-years/kWh	The ratio of the total number of jobs created in the whole lifecycle to the total amount of electricity generated
Worker injuries *(WI)*	injuries/kWh	The ratio of injuries to employees (in the whole lifecycle) to total amount of electricity generated, calculated based on employment in the whole life cycle
Public support index (*PSI*)	%	Attitude towards electricity generation options, giving an indication of net support
Diversity of fuel supply *(DFS)*	no units	Measure of energy security, indicating the dependence on foreign imports to meet energy needs

[a]DCB: dichlorobenzene; R11:tricholorofluoromethane

S3. Variations in the values of sustainability indicators across the electricity options

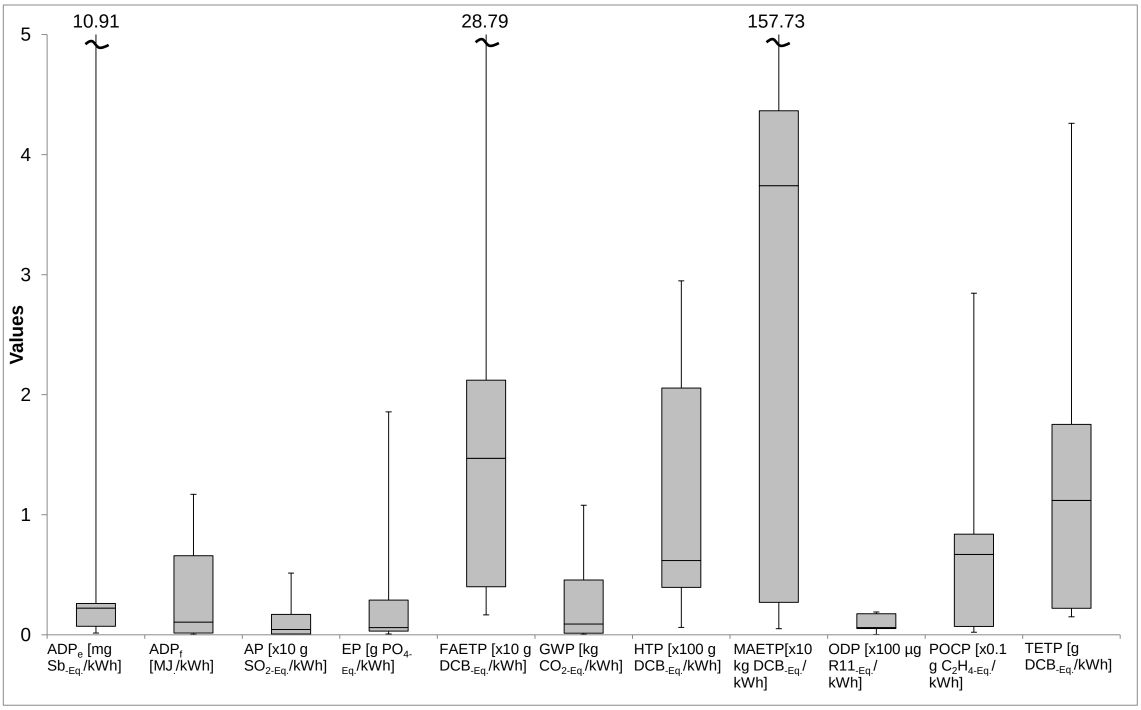

Figure S1: Variations in the environmental impacts for all nine electricity options considered in the study.

[Data obtained from Cooper et al. (2014). The box plots show median (horizontal line) and first and third quartiles (bottom and top of the boxes) of the values. The whiskers indicate the minimum and maximum values. For indicator acronyms, see Table S1].

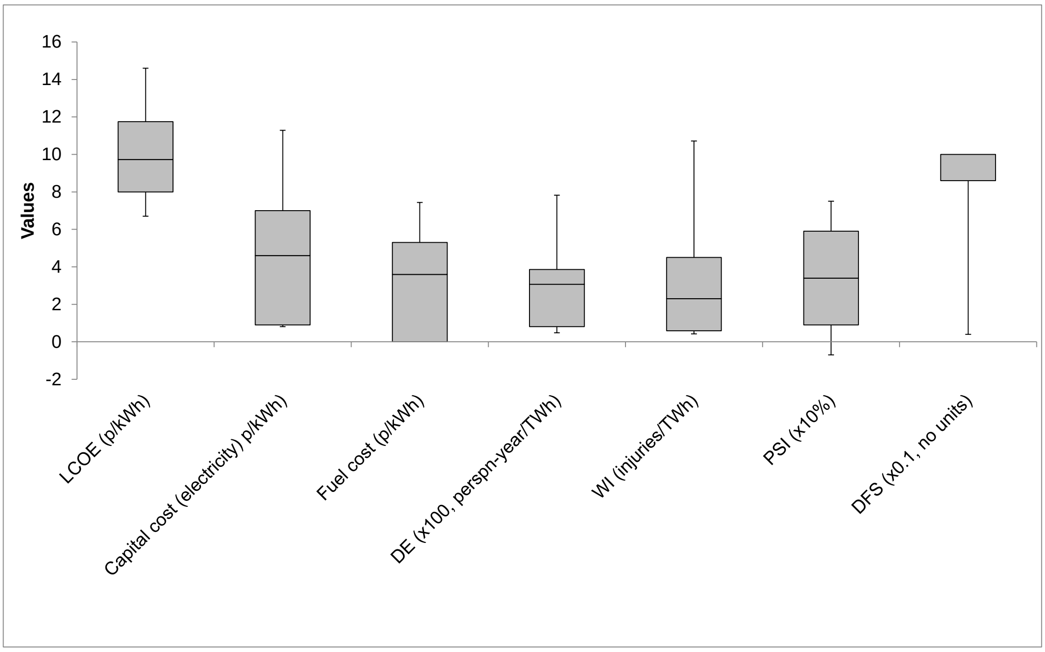

Figure S2 : Variations in the economic and social indicators for all nine electricity options considered in the study.

[Data from Cooper (2017) . The box plots show median (horizontal line) and first and third quartiles (bottom and top of the boxes) of the values. The whiskers indicate the minimum and maximum values. For indicator acronyms, see Table S1.]

S4. Changes needed in each indicator for shale gas to be comparable to other electricity options

S4.1 Comparison of shale gas with conventional gas and LNG

The environmental and social aspects need significant improvements while only a moderate reduction is needed for the economic costs. This is because shale gas has much higher environmental impacts than conventional gas and LNG (see Table 1 in the paper) but is similar in costs. For example, to be comparable with conventional gas, a 20% reduction in environmental impacts from shale gas is necessary and the environmental aspect must be 13 times more important than the other two. Alternatively, an 80% reduction in impacts should be achieved if all three aspects are considered equally important. These results correspond to the linear value function (LVF); for the exponential value function (EVF), a 100-fold reduction in environmental impacts is needed and the aspect must be three times more important. Similar results are found for LNG assuming the EVF. However, for the LVF, the required reductions in the environmental impacts are less drastic (40%) and no change in the importance of the aspect is needed (equal as the other two). By contrast, shale gas costs only need to be reduced by 10%-30% for it to compete with conventional gas and LNG.

When individual indicators are considered, reductions are needed in nine out of 11 environmental impacts for shale gas to compete with conventional gas and in eight relative to LNG. For these indicators a small reduction (6%-36%) is needed in indicators where shale gas is marginally worse than the other two gas options (Table S2). However, for the indicators where shale gas is significantly worse, 2.8-76.4 times reductions are needed. In both cases a large increase in the importance of indicators and the environmental aspect would also be needed (100-1000 times). No reductions in acidification and ozone layer depletion are needed relative to conventional gas and the required increase in their importance is also smaller than for the other indicators (ten-fold). With respect to LNG, three environmental impacts do not need improving: fossil fuels, acidification and global warming potential; however, the importance of the last must increase by 100 times, and that of the other two by ten and three times, respectively.

Improvements are also needed in social and economic indicators: 18%-31% for LCOE and fuel cost and 32% to 6.5-fold for direct employment and public support (Table S2), along with large increases in the importance of these indicators (100-1000) and their related sustainability aspects (10-100 times). For the remaining indicators, no improvements are needed, but unlike the environmental indicators, large increases in aspect/indicator importance are needed for fuel cost and diversity of fuel supply (100-10,000 times). This is due to either a marginal or no difference between the sustainability score of shale gas and the other two gas options. For worker injuries, a smaller increase in the importance of the indicator and the social aspect is needed (2-100 times) as shale gas scores better in this indicator than conventional gas and LNG.

Table S2: Improvements needed in indicators for shale gas to be comparable to conventional gas and LNG

Indicators[a]	Units	Shale gas	Conventional gas	LNG				
		Current values	*Improved values*	*Increase in importance of indicator*	*Increase in importance of related aspect*	*Improved values*	*Increase in importance of indicator[b]*	*Increase in importance of related aspect[b]*
ADP_e	mg $Sb_{-Eq.}$/kWh	0.68	0.10	1000	100	0.20	1000	100
ADP_f	MJ/kWh	6.58	6.20	1000	100	6.58	10	5
AP	g $SO_{2\text{-}Eq.}$/kWh	0.35	0.35	10	2	0.35	3	1
EP	g $PO_{4\text{-}Eq.}$/kWh	0.17	0.02	1000	100	0.02	1000	100
FAETP	g $DCB_{-Eq.}$/kWh	13.10	1.40	10,000	100	1.40	10,000	100
GWP	g $CO_{2\text{-}Eq.}$/kWh	455.78	400.00	1000	100	455.78	100	100
HTP	g $DCB_{-Eq.}$/kWh	54.30	35.00	1000	100	35.00	1000	100
MAETP	kg $DCB_{-Eq.}$/kWh	37.42	0.49	100,000	1000	0.49	100,000	1000
ODP	µg $R11_{-Eq.}$/kWh	17.30	17.30	10	10	5.90	1000	100
POCP	mg $C_2H_{4\text{-}Eq.}$/kWh	83.80	30.00	1000	100	65.00	1000	100
TETP	g $DCB_{-Eq.}$/kWh	1.70	0.14	10,000	100	0.20	1000	100
LCOE	pence/kWh	9.59	7.90	100	10	7.61	100	10
Capital cost	pence/kWh	0.81	0.81	1000	100	0.81	1000	100
Fuel cost	pence/kWh	6.51	4.80	1000	100	4.50	1000	100
Direct employment	person-yr/TWh	47.70	63.00	100	100	327.00	100	100
Worker injuries	injuries/TWh	0.42	0.42	100	100	0.42	10 (LVF)<br>100 (EVF)	2 (LVF)<br>100 (EVF)
Public support index	%	5.60	35.00	100	100	15.00	100	100
Diversity of fuel supply	-	1.00	1.00	10,000	10,000	1.00	1.5 (LVF)<br>5 (EVF)	1

[a] For the acronyms, see Table S1.
[b] LVF: linear value function; EVF: exponential value function

S4.1 Comparison of shale gas with nuclear power

As nuclear power ranks significantly better than shale gas in the base case (see Figure 1 in the paper), significant improvements and increases in the importance of sustainability aspects and indicators are needed if shale gas is to be comparable. The magnitude of the improvements and increases in importance are similar to those needed for it to compete with conventional gas as nuclear has a similar ranking to it. As shown in Table 7 in the paper, the environmental and social aspects need the largest improvements (up to 100 times) while the needed reductions in costs are smaller (25%-40%).

When the individual indicators are targeted (Table S3), improvements are needed in seven out of the 11 environmental indicators. For these, 89% to 91-fold reductions are needed along with large increases in aspect/indicator importance (100-10,000 times). For the remaining four (human, freshwater and marine toxicity and ozone layer depletion), no reductions are needed and smaller increases in aspect/indicator importance are possible (5-1000 times). For the economic and social indicators, improvements are needed in the levelised cost of electricity, fuel cost, direct employment and public support (Table S3). The levelised costs of electricity need a 21% reduction and 10-100 times increase in aspect/indicator importance, while the fuel cost must be reduced 16-fold and the importance of the aspect and the indicator should increase by 100-1000 times. However, the capital cost of nuclear power is considerably higher than that of shale gas and, as a result, no reductions in capital cost are needed and a much smaller increase in the aspect/indicator importance (2-100 times). A 72%-79% increase in direct employment and public support are required along with a 100-fold increase in the aspect and indicator importance (Table S3). No improvements in worker injuries and diversity of fuel supply are necessary for shale gas to compete with nuclear power but the aspect/indicator importance must be 3-100 times higher.

Table S3: Improvements needed in indicators for shale gas to be comparable to nuclear power

Indicators[a]	Units	Current values	Improved values	Increase in importance of the indicator[b]	Increase in importance of related aspect[b]
ADP_e	mg $Sb_{-Eq.}$/kWh	0.68	0.05	1000	100
ADP_f	MJ/kWh	6.58	0.08	1000	100
AP	g $SO_{2-Eq.}$/kWh	0.35	0.04	1000	100
EP	g $PO_{4-Eq.}$/kWh	0.17	0.02	1000	100
FAETP	g $DCB_{-Eq.}$/kWh	13.10	13.10	100	10
GWP	g $CO_{2-Eq.}$/kWh	455.78	5.00	1000	100
HTP	g $DCB_{-Eq.}$/kWh	54.30	54.30	10	10
MAETP	kg $DCB_{-Eq.}$/kWh	37.42	37.42	1000	100
ODP	µg $R11_{-Eq.}$/kWh	17.30	17.30	100	5
POCP	mg $C_2H_{4-Eq.}$/kWh	83.80	4.00	1000	100
TETP	g $DCB_{-Eq.}$/kWh	1.70	0.73	10,000	100
LCOE	pence/kWh	9.59	7.60	100	10
Capital cost	pence/kWh	0.81	0.81	3 (LVF)<br>100 (EVF)	2 (LVF)<br>10 (EVF)
Fuel cost	pence/kWh	6.51	0.40	1000	100
Direct employment	person-yr/TWh	47.70	88.00	100	100
Worker injuries	injuries/TWh	0.42	0.42	100	100
Public support index	%	5.60	10.00	100	100
Diversity of fuel supply	-	1.00	1.00	10 (LVF)<br>5 (EVF)	3 (LVF)<br>5 (EVF)

[a] For the acronyms, see Table S1.
[b] LVF: linear value function; EVF: exponential value function.

S4.3 Comparison of shale gas with hydro and biomass electricity

Hydroelectricity and biomass are the bottom ranking renewables assuming equal importance of all aspects and indicators; shale gas outranks the former for the EVF and the latter for the LVF (see Figure 2 in the paper). Therefore, improvements to shale gas to compete with hydroelectricity are only applicable to the LVF and for biomass to the EVF. As both options are closer in ranking to shale gas than conventional gas, LNG and nuclear, smaller improvements and increases in the importance of aspects and indicators are needed, as shown in Table 8 in the paper. The social aspect needs the largest improvement (8-10 times), followed by the environmental (20%-50%) and economic (20%) aspects.

However, significant improvements (9-329 times) are needed in all environmental indicators for shale gas to compete with hydroelectricity (Table S4). This is because the latter is the best option for nine out of the 11 environmental indicators and the second and third best for the other two, respectively. A 100-10,000 times increase in the importance of the aspect and the indicators is also needed. Relative to biomass, four impacts from shale gas (depletion of elements and fossil resources, global warming and ozone layer depletion) need reducing by 3.5-11.4 times, along with 100-10,000 times increase in aspect/indicator importance (Table S4). For the remaining seven indicators, no improvements are needed but the importance of the aspects and indicators must increase by 2-100 times.

For the economic indicators, shale gas has lower levelised and capital cost than both hydro and biomass electricity, but its fuel cost is higher. As a result, no reductions in levelised and capital cost are needed but an increase in aspect/indicator importance of up to 1000 times is required (Table S4). On the other hand, fuel cost must be reduced to zero and the importance of the aspect/indicator increase 10,000-fold for it to compete with hydroelectricity while a 20% reduction and a 100-fold increase in the importance is needed for it to compete with biomass.

Shale gas scores much better than both renewables for worker injuries and hence no improvement in this social sustainability indicator is needed but up to 50-fold increase in aspect/indicator importance is required. Direct employment should be improved by 16.4 times and 13 times higher public support is required for shale gas to compete with hydropower, along with a 100-fold increase in aspect/indicator importance (Table S4). As both shale gas and hydro have the maximum diversity of fuel supply no improvement is needed in this indicator, but aspect/indicator importance must be increased 10,000-fold. To compete with biomass, an eight-fold increase in direct employment and 10.4 times greater public support are needed, together with 100-1000 times increase in aspect/indicator importance. For the diversity of fuel supply, biomass scores lower than shale gas and hence no improvements are needed, but a five to 100 times increase in aspect/indicator importance is necessary.

Table S4: Improvements needed for each indicator for shale gas to be comparable to hydro and biomass electricity.

Indicators[a]	Units	Shale gas	Hydro			Biomass		
		Current values	*Improved values[b]*	*Increase in importance of indicator*	*Increase in importance of related aspect*	*Improved values[c]*	*Increase in importance of indicator*	*Increase in importance of related aspect*
ADP_e	mg $Sb_{-Eq.}$/kWh	0.68	0.01	10,000	100	0.13	10,000	100
ADP_f	MJ/kWh	6.58	0.02	10,000	100	0.60	10,000	100
AP	g $SO_{2-Eq.}$/kWh	0.35	0.01	10,000	100	0.35	10	10
EP	g $PO_{4-Eq.}$/kWh	0.17	0.02	1000	100	0.17	5	5
FAETP	g $DCB_{-Eq.}$/kWh	13.10	1.40	10,000	100	13.10	100	100
GWP	g $CO_{2-Eq.}$/kWh	455.78	3.00	10,000	100	40.00	10,000	100
HTP	g $DCB_{-Eq.}$/kWh	54.30	5.90	10,000	100	54.30	10	3
MAETP	kg $DCB_{-Eq.}$/kWh	37.42	0.50	100,000	1000	37.42	100	10
ODP	µg $R11_{-Eq.}$/kWh	17.30	0.21	10,000	100	5.00	10,000	100
POCP	mg $C_2H_{4-Eq.}$/kWh	83.80	1.80	10,000	100	83.80	10	6
TETP	g $DCB_{-Eq.}$/kWh	1.70	0.18	10,000	100	1.70	2	3
LCOE	pence/kWh	9.59	9.59	5	5	9.59	5	2
Capital cost	pence/kWh	0.81	0.81	2	1	0.81	1000	100
Fuel cost	pence/kWh	6.51	0.00	10,000	10,000	5.20	100	100
Direct employment	person-yr/TWh	47.70	783.00	100	100	386.00	1000	100
Worker injuries	injuries/TWh	0.42	0.42	2	1	0.42	50	5
Public support index	%	5.60	73.00	100	100	58.00	1000	100
Diversity of fuel supply	-	1.00	1.00	10,000	10,000	1.00	100	5

[a] For the acronyms, see Table S1.
[b] For linear value function only.
[c] For exponential value function only.

S5. Pedigree matrix

Table S2: Pedigree matrix characteristics and criteria used to grade data quality (Althaus et al., 2007; Weidema et al., 2013).

	Score				
**Criteria**	1	2	3	4	5
Reliability	Published data based on measurements	Published data partially based on assumptions *Or* Non-published data based on measurements	Non-published data based on estimates	Estimates verified by experts	Non-verified estimates
Completeness	Representative of all relevant sites for market considered, over an adequate time period	Representative of ~50% relevant sites for market considered, over an adequate time period	Representative of <50% relevant sites for market considered, over an adequate time period *Or* Representative of >50% relevant sites for market considered, short time period	Representative of only one site relevant for market considered, over an adequate time period	Representativeness unknown
Temporal correlation	≤ 3 years difference between data and study	≤ 6 years difference between data and study	≤ 10 years difference between data and study	≤ 15 years difference between data and study	≥3 years difference between data and study, or age unknown
Geographical correlation	Area of study	Larger area including study area	Similar area	Slightly similar area	Unknown or distinctly different area
Further technological correlation	Data for technology from company/operator	Data not from company/operator but for same technology	Data on similar processes and materials but different technology	Data on similar processes and materials	Data on similar processes and material but laboratory scale
Sample size	> 100 measurements	> 20 measurements	> 10 measurements	≥ 3 measurements	Unknown

S6. Data quality assessment

Table S3: Pedigree matrix results for data sources.

Ecoinvent LCI data (LCA only)[a]		Reliability	Completeness	Temporal correlation	Geographical correlation	Technological correlation	Sample size	Total
Shale gas		1	1	3	2	2	1	10
Conventional gas		1	1	4	2	2	1	11
Liquefied natural gas		1	1	4	2	2	1	11
Coal		1	2	4	2	3	1	13
Nuclear		1	1	5	3	2	1	13
Hydro		1	2	4	2	2	1	12
Solar PV		1	1	3	2	2	1	10
Wind		1	1	4	3	2	1	12
Biomass		1	2	4	4	3	1	15
**Literature LCI data[a]**	**No. of data sources**	**Reliability[b]**	**Completeness[b]**	**Temporal correlation[b]**	**Geographical correlation[b]**	**Technological correlation[b]**	**Sample size[b]**	**Total**
LCA – overall data quality (average)		***3.06***	***1.25***	***1.26***	***1.8***	***1.75***	***4.7***	***13.82***
Shale gas extraction	8	1.63	2.25	1.63	5.00	1.75	3.50	15.76
Power plant	1	4.00	1.00	1.00	1.00	2.00	5.00	14.00
Conventional gas	1	4.00	1.00	1.00	1.00	2.00	5.00	14.00
Liquefied natural gas	3	1.67	1.00	1.67	1.00	1.00	5.00	11.34
Power plant (other electricity options)	1	4.00	1.00	1.00	1.00	2.00	5.00	14.00
LCC – overall data quality (average)		***2.17***	***1.00***	***1.08***	***1.00***	***1.83***	***5.00***	***12.08***
Capital cost	5	2.00	1.00	1.00	1.00	2.00	5.00	12.00
Operating cost	2	2.00	1.00	1.00	1.00	2.00	5.00	12.00
Labour	2	2.00	1.00	1.00	1.00	2.00	5.00	12.00
Community charter	1	3.00	1.00	1.00	1.00	1.00	5.00	12.00
Power plant costs	2	2.00	1.00	1.50	1.00	2.00	5.00	12.50
Levelised cost (other electricity options)	1	2.00	1.00	1.00	1.00	2.00	5.00	12.00

Literature LCI data[a]	No. of data sources	Reliability[b]	Completeness[b]	Temporal correlation[b]	Geographical correlation[b]	Technological correlation[b]	Sample size[b]	Overall
Social sustainability assessment – overall data quality (average)		***1.36***	***1.04***	***1.44***	***2.37***	***1.52***	***3.71***	***11.43***
Public support	7	1.57	1.57	1.29	2.14	1.29	2.14	10.00
Worker injury	3	1.00	1.00	1.33	1.00	1.33	5.00	10.66
Direct employment	19	1.74	1.00	1.84	1.42	1.56	5.00	12.56
Local employment	2	1.50	1.00	1.00	3.00	2.00	3.50	12.00
Diversity of fuel supply	8	1.50	1.00	1.00	1.38	1.13	5.00	11.01
Noise	3	1.00	1.00	1.00	3.67	1.67	5.00	13.34
Traffic	1	2.00	1.00	2.00	5.00	2.00	5.00	17.00
Land use conflict	1	1.00	1.00	1.00	1.00	1.00	1.00	6.00
Wastewater volume	2	1.50	1.00	1.00	5.00	2.00	3.00	13.50
Wastewater treatment	3	1.33	1.00	1.33	3.67	2.00	2.67	12.00
Media bias	5	1.00	1.00	1.00	1.00	1.00	1.00	6.00
Regulation (US)	3	1.00	1.00	2.00	5.00	2.00	1.00	12.00
Regulation (UK)	1	1.00	1.00	2.00	1.00	1.00	5.00	11.00
Gender equality	3	1.67	1.00	1.67	1.67	1.33	5.00	12.34
Spending on local supplies	1	2.00	1.00	1.00	1.00	2.00	5.00	12.00
Direct community investment	4	1.00	1.00	2.50	1.00	1.00	5.00	11.50
Future scenarios – overall data quality		***1.88***	***1.00***	***1.75***	***1.47***	***1.03***	***5.00***	***12.13***
Gas mix	1	2.00	1.00	2.00	2.00	1.00	5.00	13.00
Electricity mix	2	2.00	1.00	2.00	1.50	1.00	5.00	12.50
Levelised cost (other electricity options)	1	2.00	1.00	2.00	1.00	1.00	5.00	12.00
Diversity of fuel supply	8	1.50	1.00	1.00	1.38	1.13	5.00	11.01

[a] LCI: life cycle inventory; LCA: life cycle assessment.
[b] Average values for the number of data sources used.

References

Althaus, H.-J., Doka, G., Dones, R., Heck, T., Hellweg, S., Roland Hischier, Nemecek, T., Rebitzer, G., Spielmann, M. and Wernet, G. **2007**. *Overview and methodology: Data v2.0 (2007)*. *The ecoinvent Centre*. Zurich, CH. Retrieved from: http://www.ecoinvent.org/database/older-versions/ecoinvent-version-2/methodology-of-ecoinvent-2/methodology-of-ecoinvent-2.html.

Cooper, J. **2017**. *Life cycle sustainability assessment of shale gas in the UK*. PhD thesis, The University of Manchester.

Cooper, J., Stamford, L. and Azapagic, A. **2014**. Environmental impacts of shale gas in the UK: Current situation and future scenarios. *Energy Technology*, 2, 1012-1026.

Weidema, B. P., Bauer, C., Hischier, R., Mutel, C., Nemecek, T., Reinhard, J., Vadenbo, C. O. and Wernet, G. **2013**. *Overview and methodology: Data quality guideline for ecoinvent database version 3 (final)*. *The ecoinvent Centre*. Zurich, CH. Retrieved from: https://www.ecoinvent.org/files/dataqualityguideline_ecoinvent_3_20130506.pdf.
